# Impact of medicine shortages on patients - a framework and application in the Netherlands

**DOI:** 10.1186/s12913-022-08765-x

**Published:** 2022-11-17

**Authors:** Doerine J. Postma, Peter A. G. M. De Smet, Kim Notenboom, Hubert G. M. Leufkens, Aukje K. Mantel-Teeuwisse

**Affiliations:** 1 Division Pharmacoepidemiology & Clinical Pharmacology, Utrecht Institute for Pharmaceutical Sciences (UIPS), Utrecht University, PO Box 80 082, 3508 TB Utrecht, The Netherlands; 2 Royal Dutch Pharmacists Association, The Hague, the Netherlands; 3 Departments of IQ healthcare and of clinical pharmacy, Radboud University Medical Centre, Radboud Institute for Health Sciences, Nijmegen, the Netherlands; 4 Dutch Medicines Evaluation Board, Utrecht, the Netherlands

**Keywords:** Medicine shortages, Impact, Framework

## Abstract

**Background:**

Medicine shortages are often described in plain numbers, suggesting all shortages have a uniform impact. However, some shortages have a direct and serious effect on patients and need a prompt reaction from stakeholders. This study aims to create a broad framework to assess the impact of a shortage.

**Method:**

We identified high impact shortages and selected exemplary shortages which we considered our learning cases. From five learning cases, we identified elements that had a potentially profound impact on one or more of these cases. We tested data saturation on the elements with another five test cases. Based on these elements, we created a framework to assess impact of shortages on patients and presented practical examples how to rate these different elements. Subsequently, we visualised the impact of these five learning cases on patients in radar charts.

**Results:**

The five elements which we identified as potentially having a large impact were 1) alternative product, 2) disease, 3) susceptibility, 4) costs and 5) number of patients affected. The five learning cases rated high on different elements, leading to diverse and sometimes even opposite patterns of impact.

**Conclusion:**

We created a framework for assessing the impact of a medicine shortage on patients by means of five key elements. By rating these elements, an indication of the impact can be obtained.

**Supplementary Information:**

The online version contains supplementary material available at 10.1186/s12913-022-08765-x.

## Background

Medicine shortages are increasing all over the world [1, 2]. These shortages have worsened during the COVID-19 pandemic affecting many patients globally. Patients with COVID-19 are affected due to an increased demand and stockpiling of medicines which are essential for COVID-19 treatment such as midazolam and propofol for sedation of intubated patients. Also, patients not suffering from COVID-19 who depend on medicines that also show promise for treating COVID-19 are affected due to increased demand. For instance, patients with systemic lupus erythematosus face difficulties obtaining their treatment of hydroxychloroquine [3].

Patients who need medicines that are unrelated to COVID-19 are bound to become affected as well since the pandemic has limited the manufacturing and exporting of active substances and medicines worldwide [4]. China is the world’s leading producer and exporter of active pharmaceutical ingredients by volume. As factories in China closed to mitigate the impact of the COVID-19 virus, less active pharmaceutical ingredients were manufactured [5]. India, the world’s leading supplier of generic medicines, put a ban on the export of medicines such as paracetamol, several antibiotics and hydroxychloroquine. After a month, this ban was partially lifted, when the availability of these medicines for its own population was assured [6].

The unavailability of medicines can impact patients. According to a definition by the Organisation for Economic Co-operation and Development (OECD), impact can be positive or negative; intended or unintended [7]; directly or indirectly. For the unavailability of medicines, direct and indirect impact can be tangible. The direct impact renders patients unable to prevent and treat their diseases. This inability can cause harm to patients [8]. Patients experience indirect impact when regulatory authorities and healthcare professionals have less time for other regulatory and care giving tasks, respectively [9–11]. These professionals also feel threatened by their inability to fulfil their moral obligations towards patients and society [12]. In general, society may be confronted with increased healthcare expenditures, such as extra medicine and personnel costs, resulting in increased public funding [13].

Shortages are often described in plain numbers, suggesting all shortages have a uniform impact. However, some shortages have a large impact and are less ‘forgiving’ than others; they need a prompt reaction from stakeholders to avoid patient harm, extra costs, or both. The term ‘forgiveness’, which comes from the field of patient adherence, is related to the number of medication doses that a patient can miss without negatively impacting their treatment outcome [14]. Missing too many doses can directly harm the patients. Similarly, shortages have degrees of ‘forgiveness’. Stakeholders such as authorities and healthcare professionals have their own responsibilities, resulting in divergent assessments of impact. Also their perspectives differ regarding shortages [15]. For example, authorities and marketing authorisation holders (MAHs) focus on the impact of shortages on a population-wide level, whereas healthcare professionals and patients tend to focus on the effects of shortages at an individual level. To quantify the ‘true’ impact of shortages on patients has shown to be difficult with these different perspectives [16].

Several publications have described the impact of shortages with or without further specification of impact [17–19]. To assess the impact in a more qualitative way, the Parenteral Drug Association (PDA) published a risk-based approach [20] from medicine industry’s perspective. In this approach, both the absence of the prescribed treatment and the presence of an alternative treatment are classified as high, medium or low risk. This risk level, along with the likelihood of a shortage, determines the priority level. Although this was a patient-centred approach, the decision model includes limited aspects of direct and indirect impacts on patients. Other aspects may include the risk of medication errors [8], ease of switching [21] and financial consequences [22, 23].

The COVID-pandemic has shown us the vulnerability of the full supply chain and the increased risk of shortages as a result. Thus, it is crucial to assess a shortage’s direct and indirect impact quickly and in a more qualitative way. The aim of our study was therefore to create a broad framework with different perspectives in mind to assess the impact of shortages, covering both the direct and indirect impact on patients. This framework may help to identify the core drivers behind the impact for specific shortages and to obtain an indication of the degree of forgiveness of a shortage.

## Method

### Study type and design

The design of this study was qualitative, observational, retrospective and descriptive. We evaluated possible shortages which were signaled in the Netherlands. A purposive sampling strategy was applied to select discriminative unforgiving shortages which led to a prompt reaction from stakeholders: our learning cases. We expected the core drives behind patient impact to clearly stand out for these shortages, giving us insight into direct and indirect patient impact of medicine shortages.

We outlined these exemplary learning cases in detail in case reports, which allowed us to fully explore the aspects of the shortages in a narrative way. Follow up for these narratives ended 31 December 2017. From these case reports, we went into further detail on the discriminative elements of patient impact; we elaborated on the elements in a non-specific way.

To test whether the identification of the elements was complete and data saturation was reached, we randomly selected another set of five cases from the sample of the overlapping shortages with high impact. We regarded them as our test cases. If new elements were identified, another sample of five cases was tested to assure completeness of the discriminative elements.

### Study setting

**Fig. 1:**
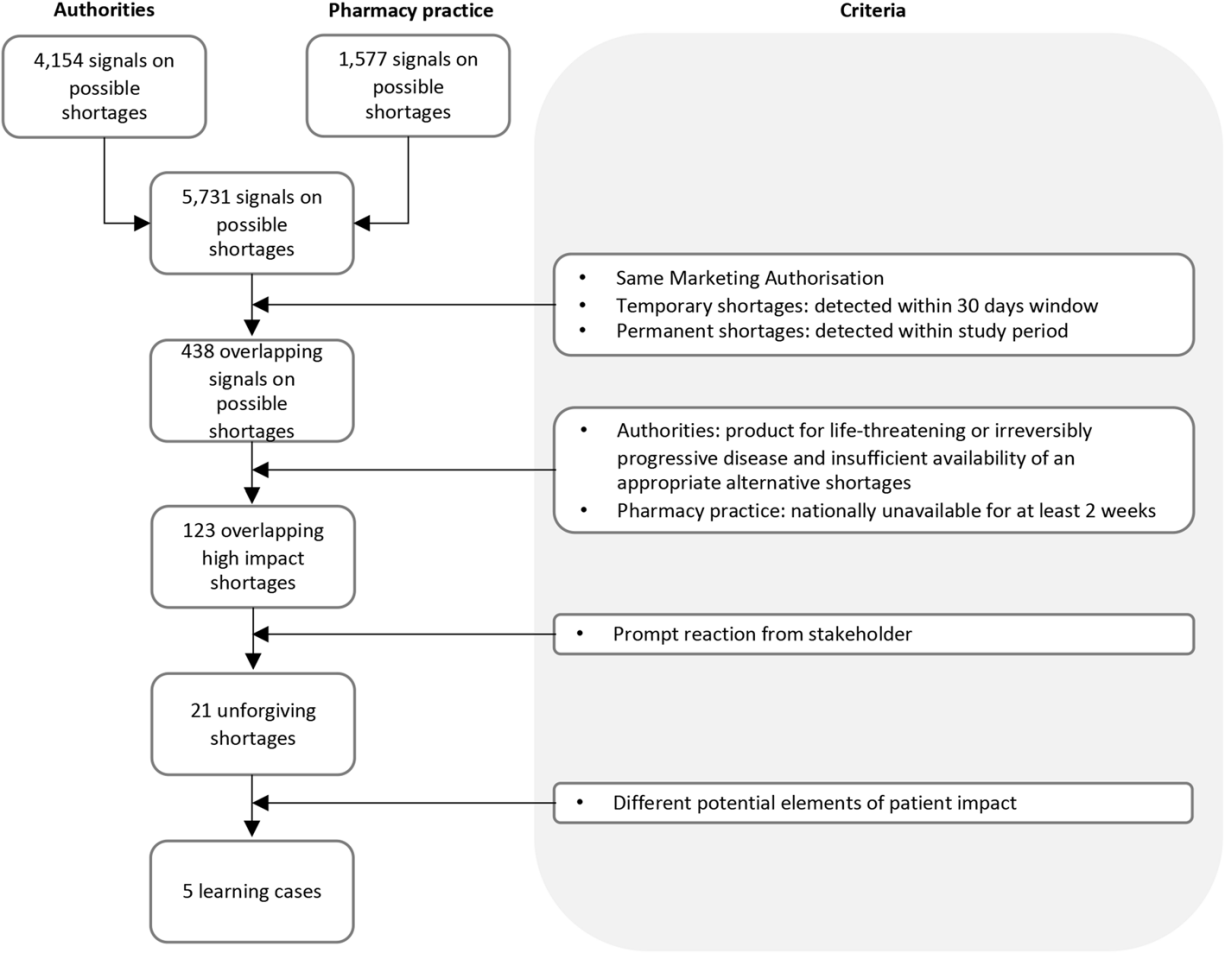
Selection of learning cases from signals on potential shortages

We evaluated possible shortages which were signaled by the authorities (Medicines Evaluation Board [MEB] and Health and Youth Care Inspectorate) or the pharmacy practice (KNMP Farmanco) in the Netherlands (Fig. 1). A total of 5731 signals on potential shortages were detected by the authorities (*n* = 4154) and the pharmacy practice (*n* = 1577) between 1 January 2012 and 31 December 2015 as described in more detail in previous research [24].

### Sampling technique

From the total of 5731 signals only 438 signals on possible shortages overlapped. Since these overlapping signals were detected by both stakeholders, we regarded them as having a potential high impact on patients. Overlapping signals were defined as signals for the same marketing authorization within a specified timeframe. For temporary shortages, the signal needed to be received at both institutions within a 30-day time period. For permanent shortages, the signal needed to be received within the study period.

To ensure that we based our framework on actual shortages versus possible shortages, we determined the status of the shortages using the criteria set forth by the authorities and the pharmacy practice. For authorities, this meant that the medicinal product needed to treat a life-threatening or irreversibly progressive disease and there was insufficient availability of an appropriate alternative. According to pharmacy practice, it meant that the medicinal product needed to be nationally unavailable for at least 2 weeks. We retrieved the final number of 123 shortages that met these criteria, which served as the basis for further analyses.

Two experts on medicine shortages screened the 123 overlapping shortages with high impact together to select unforgiving shortages which led to a prompt reaction from stakeholders. Consensus between the two experts was achieved by discussing prompt reactions such as extensive effort by authorities or healthcare professionals to mitigate the impact of the shortage on patients or generating media attention by patients. For these selected unforgiving shortages (*n* = 21), we expected elements potentially having a high impact on patients to clearly stand out. The experts subsequently selected shortages by consensus with different elements limiting the selection to one case per similar reaction. Five discriminative shortages remained, and they emerged as our learning cases.

### Data analysis

Descriptive statistics were used to present the direct and indirect impact of shortages, by analysing how the five learning cases rated on the different elements by applying our framework. Finally, we visualised the impact of the elements using a radar chart which was created in Microsoft Excel version 16.34.

## Results

Our learning cases were Bacillus Calmette-Guérin (BCG) instillation, doxycycline tablets, epinephrine auto-injector, levothyroxine tablets and penfluridol tablets. Full details on these cases are described in Supporting Information 1, S1.

### Elements for a framework to assess impact

We identified elements which had a large patient impact on one or more of these five learning cases, making the shortages unforgiving. The elements were 1) alternative product, 2) disease, 3) susceptibility, 4) costs and 5) number of patients affected. These elements represent perspectives from different stakeholders. Table 1 elaborates on these elements in a general, non-country-specific way. For three of the elements, alternative product, susceptibility, and costs, we distinguished two aspects per element.

**Table 1:** Examples of elements influencing the impact of a medicine shortage

Impact	Alternative product		Disease (disability weight)	Susceptibility		Costs		Number of patients affected (% of population)
	*primary aspects*	*secondary aspects*		*vulnerability*	*trust in alternative therapy*	*to patients*	*to society*	
**Low**	- same substance, licenced, on-label	- same regimen, strength, concentration or instruction for use - different excipients or labelling	0–0.058	- 19 - 75 years	full trust	full reimbursement	no extra medicines costs and/or personnel costs	< 0.05
**Medium**	- different substance, licenced, on-label - same substance, unlicenced, on-label - different substance, licenced, off-label - different substance, unlicenced, on-label - different substance, unlicenced, off-label	- interchangeable with extra control - different regimen, strength, concentration or instruction for use or storage	0.058–0.224	- 2 – 18 years, - pregnant and nursing women - patients with divergent metabolism	moderate trust	additional payment	minor to moderate extra medicines costs and/or personnel costs	0.05–0.5
**High**	- no or inferior therapy available	- different route of administration	0.224–1	- < 2 years - > 75 years - patients depending on social care	no trust	full payment	high extra medicines costs and/or personnel costs	> 0.5

We ranked the examples for each of the five elements with increasing impact and colour coded them for visualisation purposes: green for low rate, orange for moderate rate and red for high rate. We used these colours instead of a number scale to show the indicative character since we did not perform a quantitative analysis.

### Test cases for data saturation in identification of elements

We randomly selected five cases from the sample of the 123 shortages as test cases to check whether data saturation in the identification of the elements was reached. Our test cases were benzylpenicillin, dactinomycin, ketoprofen, pentazocine and topiramate. We constructed narratives for these shortages, and no new elements were identified based on these narratives. Therefore, we considered data saturation to be reached and the range of elements to be complete. From the test cases, we were able to enrich the aspects of the elements. One of the test cases was an antibiotic, and the alternative product was unwanted due to the risk of microbiological resistance. This was an enrichment of a secondary aspect. For the learning case of the antibiotic doxycycline, this aspect was not profound since, in general, doxycycline is only occasionally prescribed to patients. However, the antibiotic from the test case, benzylpenicillin, is prescribed frequently to patients, resulting in a higher risk of microbiological resistance.

### Application of the proposed framework to the learning cases

To determine the direct and indirect impact shortages have on patients, we applied our framework to the learning cases for the Dutch situation. Full details on these cases are summarised in Table 2 (see also Supporting Information 1, S1).

**Table 2:** Rates on elements influencing the impact of medicine shortages for learning cases

Learning case	Alternative product		Disease (disability weigth)	Susceptibility		Costs		Number of patients affected (% of population)
	*primary aspects*	*secondary aspects*		*vulnerability*	*trust in alternative therapy*	*to patients*	*to society*	
BCG instillation	no equivalent treatment available	different regimen	0.288	> 75 years	no trust	fully reimbursed	no extra costs	0.03
doxycycline tablets	different substance licenced on-label	same regiment and use	0.051	19–75 years	full trust	fully reimbursed	extra medicines costs and personnel costs	0.71
epinephrine auto-injector	same substance and licenced	different instruction	1	3–9 years 19–75 years	moderate trust	fully reimbursed	extra personnel costs	0.35
levothyroxine tablets	same substance, licenced, on-label,	interchangeable with extra control	0.019	19–75 years	no trust	additional payment	no extra costs	2.06
penfluridol tablets	no equivalent treatment available	different regimen	0.558	depend on social care	no trust	fully reimbursed	extra medicines costs	0.08

**Fig. 2:**
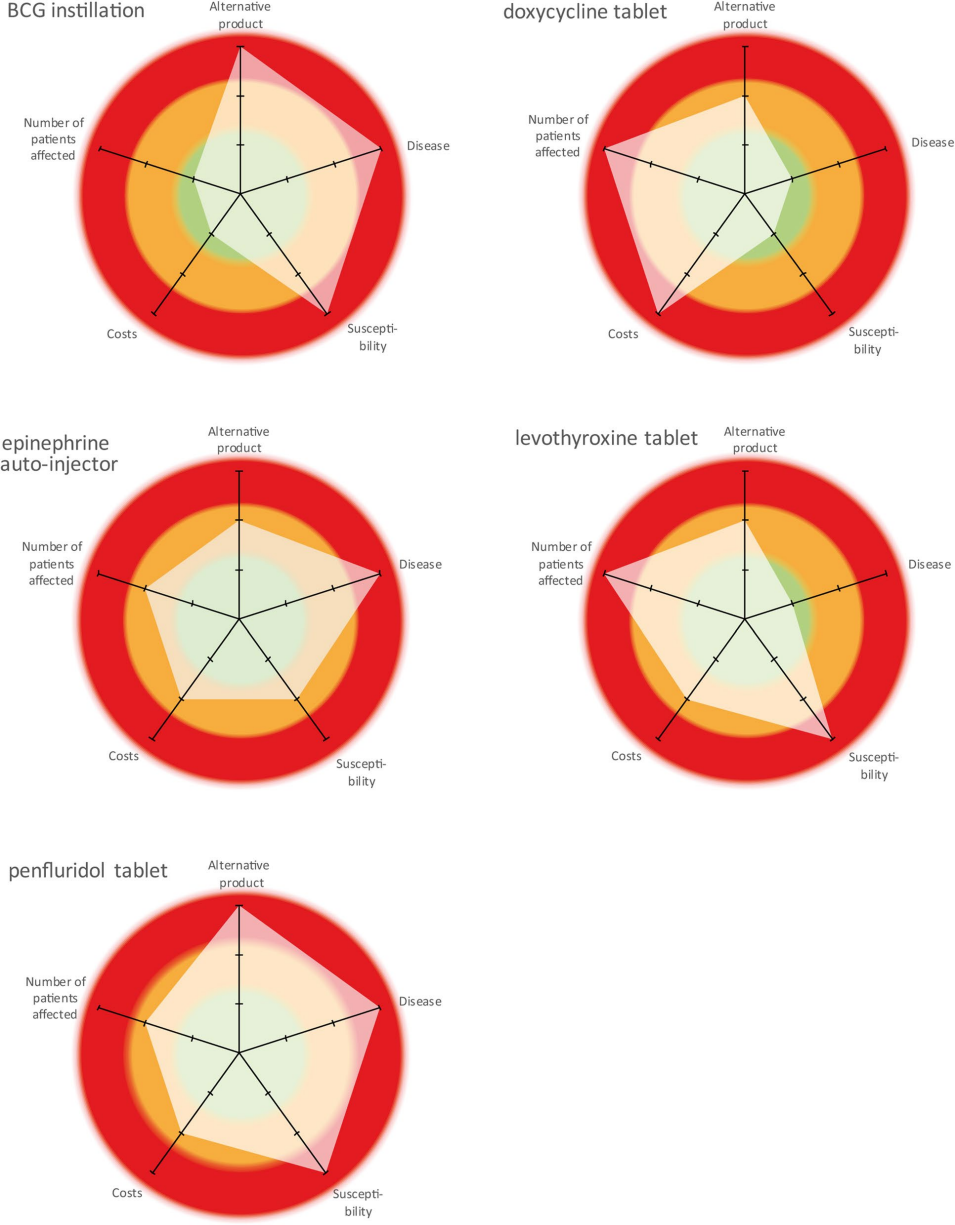
Rated elements influencing impact of medicine shortages for learning cases. Green = low rate, Orange = moderate rate, Red = high rate

We translated the elements summarised in Table 2 into radar charts with corresponding colours to visualise the impact of the shortages (Fig. 2a-e). If two items within one element were rated at a different level, the highest impact was considered to be the overall rate for that element.

- BCG instillation: no equivalent treatment available; disability weight 0.288; no trust in alternative therapy; no extra costs; 0.03% of population affected.
- doxycycline tablet: alternative product is a different substance; disability weight 0.051; patient population is not susceptible; extra costs for medicines and personnel; 0.71% of population affected.
- epinephrine auto-injector: alternative product has different instructions for use; disability weight 1; moderate trust in alternative therapy; extra costs for personnel; 0.35% of population affected.
- levothyroxine tablet: alternative therapy is interchangeable with extra control; disability weight 0.019; no trust in alternative therapy; extra costs for patients; 2.06% of population affected.
- penfluridol tablet: no equivalent therapy available; disability weight 0.558; patients are vulnerable and have no trust in alternative therapy; extra costs for medicines; 0.08% of population affected.

All cases were rated high for at least one of the elements – alternative product, disease, susceptibility, costs or the number of patients affected. The element(s) with the highest rate, however, differed across our learning cases. Several of the other elements of these cases were rated in the high or moderate category. Three of the five shortages also had the lowest rate for one or more elements. The radar charts clearly depict opposite patterns for shortages of BCG instillation and penfluridol tablets versus doxycycline tablets.

## Discussion

Our study shows that unforgiving shortages can differ on elements that have a large effect on patient impact. We identified five key elements, namely alternative product, disease, susceptibility, costs, and number of patients affected. These elements led to the development of a framework to systematically assess the impact of medicine shortages on patients and visualise the impact using radar charts. Different patterns observed in these charts indicate that different elements can be the key driver(s) behind the impact of medicine shortages and key contributors to the unforgiveness of the shortage. The results of this study suggest that unforgiving shortages of a particular medicine are rated with a high or moderate score on multiple elements.

### Alternative product

The availability of the alternative product and its characteristics may have a profound impact on the clinical effects and the risk of medication errors. The impact on patients is not only determined by its primary aspects, such as active substances, licencing and on- and off-label use, but also by its secondary aspects, such as different routes of administration, strengths, concentrations or excipients, and the possibility of extra controls to guide therapy in switching patients. Whereas primary aspects are apparent, secondary aspects are less apparent but also relevant. The most evident primary aspect to patients, healthcare professionals and authorities is the active substance. An alternative product may contain the same or a different active substance. The worst-case scenario is the absence of an adequate alternative therapy. In addition to the substance, we distinguish between licenced and unlicenced products. Licenced products have been verified to meet acceptable standards of efficacy, safety and quality and are manufactured to appropriate quality standards according to national authorities. Examples of unlicenced products not verified by national authorities are pharmacy preparations and imported products. We also distinguish between on-label and off-label use, since the clinical evidence and the safety profile differ. On-label use is use within the terms of the licence (including indication and age-group) and off-label use is outside of it.

A shortage is preferably solved by on-label use of a licenced alternative product that contains the same active substance – called a generic substitution. This way, the same clinical effect and safety profile (including side effects) is achieved, and the same quality standards apply. Whereas healthcare professionals often consider this a very low-impact solution, patients frequently have negative perceptions about switching [25]. Substances with a narrow therapeutic window may need additional follow-up after the switch, and they, therefore, create a higher impact.

Stakeholders may have different opinions on the preferred solution. In a situation where no generic alternatives are available on the market, patients may, for example, prefer an unlicenced product with the same substance as the original therapy. In contrast, authorities may prefer the on-label use of a licenced product with a different substance – called a therapeutic substitution. When an alternative product with a different substance is used, prescribers may be less familiar with the alternative therapy. Therefore, healthcare professionals should be alert about its potentially different adverse effects on patients and its contraindications [26].

The secondary aspects – such as different routes of administration, strength, concentration or excipients – may be less obvious but still important for determining the ease and safety of switching to another product. Switching requires an initial check for safety issues [26, 27]. Differences such as other excipients, labelling or taste need to be dealt with at the start of the therapy. In contrast, other differences need attention in daily practice since they may play a role continually. For example, a healthcare professional needs to be aware if injection has a different strength, since injections are usually administered by the number of millilitres. These differences can easily lead to medication errors that may have a direct and high impact on patients. Differences in strength and concentration are the greatest risks for medication errors since they may lead to changes in dose regimen, preparation before use or instructions for use [26]. A different instruction for use, such as a different storage condition, can have a significant impact. This different storage condition could, for example, result in incorrect storage of the product, which may affect the product’s quality. It may also result in a different physical storage place, which may lead to the incorrect assumption that the product is absent. If the original product is stored at room temperature while the alternative is stored in the refrigerator, one may assume the product absent. A product with a different route of administration is usually considered an inequivalent treatment for practical reasons. For instance, a switch from an intravenous injection to an oral preparation would not be an option for patients who have severe difficulties swallowing. Conversely, an intravenous injection would not be an option for outpatients who have to administer the medicine in the absence of healthcare professionals.

The alternative product is not a static element; rather, it can change over time due to its actual availability and the duration of the shortage. The actual availability of the alternative product depends on the capability of its manufacturer to respond to the increased demand for the duration of the shortage. If this capability is insufficient, the availability of the alternative product can change over time. For instance, a generic substitution may be possible during a short period of time, whereas a therapeutic substitution may be necessary during a longer period of time. Also, the duration of the shortage itself may be lengthened, which may result in insufficient availability of the alternative product over time.

### Disease

The consequences of the absence of a medicinal product are determined by the disease, ranging from causing fatality for a life-supporting product to causing mere inconvenience for a lifestyle product. The impact of a disease can be defined in terms of ‘burden of disease’ or ‘disability-adjusted life years’. These terms are only known for a restricted number of diseases, and calculating them is time-consuming [28, 29]. We chose to use disability weights to quantify health levels associated with non-fatal outcomes. These weights have the advantage of being socially derived values based on how the majority of people perceive living with a disease or condition for a one-year period. Disability weights rate optimal health as 0 and death as 1. These weights have been published for 235 unique health states and cover the majority of diseases [30].

No threshold was found for a disability weight to have a profound impact on patients. Therefore, we determined the intertertile range of the disability weights. We set the lower tertile as our threshold for low impact of the disease and the upper tertile for high impact of the disease.

### Susceptibility

Some patients may be affected more than others when confronted by alternative treatments. Alternative treatments may impact patients clinically, psychologically, or both. The impact on vulnerable populations should be taken into consideration in the overall assessment of a shortage, according to the EMA [21]. However, there is no precise definition of when a population is vulnerable. For clinical trials, children, elderly, pregnant and nursing women are considered vulnerable [31]. Healthcare professionals also consider vulnerable populations those who have difficulty protecting themselves from harm and abuse and in promoting their own interests [32]. These populations have two aspects in common. First, they have potential age-related changes in pharmacokinetics and pharmacodynamics of medicines. Second, they may have difficulties taking care of themselves [32, 33]. Elderly persons may experience a different pharmacological and clinical effect with large interindividual differences [34]. Elderly persons are not clearly specified by age. The age of 75 years has been suggested [33], and studies have been performed on patients 75 years and older [35]. Children (up to 18 years of age) may metabolise medicines differently and may need specific attention if an alternative therapy is considered. Children younger than 2 years need extra attention since most pharmacokinetic pathways are not yet mature, so an alternative therapy can pose a substantial risk [36]. Furthermore, adults with divergent pharmacokinetics and pharmacodynamics of medicines, due to, for instance, renal impairment, hepatic impairment, or both, are also vulnerable to alternatives because of the required adjustment to dosages [37]. Changes in the pharmacology of medicines need to be dealt with once before the start of the therapy; therefore, an initial check on this aspect is advised. Since this aspect is age-related, certain age groups can be considered vulnerable. The second aspect that these populations share is the inability to take care of themselves. Children older than 12 years of age are assumed to take responsibility for their own health and medication [36], resulting in a less significant impact on them than younger children. Independent of age, however, patients who are unable to take care of themselves and those who, for instance, rely on social care need extra attention when an alternative treatment is required.

Besides pharmacological patient vulnerability, there is also psychological/social patient vulnerability. For example, lack of trust may lead to patient non-adherence to the alternative treatment, thus influencing the impact of the shortage [38]. Mistrust can be intensified by several factors. Factors that need attention, especially during a shortage, are the patients’ beliefs and concerns, conflicting medical information, and mental health. Patients’ beliefs can be very strong and are based, for instance, on information from general media. This information can emphasise the risks and side effects of the alternative treatment. Patients’ concerns may be related to the effect the alternative treatment has on their social life. For instance, patients may receive another diuretic which may cause frequent urination, thus keeping them closer to home. Conflicts in medical information in the case of a shortage may happen if information in patient leaflets differs between the product in shortage and the alternative. For example, if the patient leaflet of the product in shortage has no information on administration, while the alternative product should not be administered with milk. Finally, patients receiving treatment for mental health problems need attention when switching due to the risk of non-adherence. This includes patients with a psychiatric diagnosis, a life-threatening disease or forgetfulness. Psychiatric patients are well-known for their reluctance to accept an alternative therapy [39, 40]. Patients suffering from a life-threatening disease have a clear understanding of their current medication’s benefits and may hesitate to switch because they are afraid of their condition worsening.

### Costs

Extra costs can be assigned to the patients or to society in general. Extra costs for patients may result in the unaffordability of the alternative therapy [41]. Extra costs for society include higher prices of alternative medicines as well as personnel costs for additional work. This work is caused by (extensive) communication of healthcare professionals with other healthcare professionals and patients. For example, a pharmacist reporting to a primary care physician that a medicine is not available and suggesting an alternative treatment. Or a pharmacist explaining to a patient that only an alternative strength is available with a corresponding number of tablets. Extra medicine costs were studied to try to quantify the effect. A study in U.S. hospitals in 2010 showed an average increase in the price of 11% [13], while a survey in U.S. hospitals in 2015 revealed increased prices of 300 to 500% [42]. Extra personnel costs have only been surveyed for medicine shortages in general. Recent studies estimated extra personnel required in pharmacies to manage shortages ranged from 0.4 hours per week in Flemish community pharmacies [43] and 12.8 hours per week in European hospitals [44] to 20–40 hours per week in U.S. healthcare pharmacies [42]. These costs have not been studied per shortage. A few publications studied the costs of specific shortages but excluded personnel costs [45–47]. For our framework, costs can be based on historical costs, actual costs as well as forecasting costs using financial or economic modelling [48, 49].

### Number of patients affected

In case of a shortage, the number of patients affected should be known in order to determine if there is a sufficient supply of the alternative therapy. The number of patients affected also influences the impact of shortages on a population-wide level. If more patients are affected, then there may be higher levels of public concern. The Dutch levothyroxine crisis in 2016 involved 350,000 patients, about 2% of the Dutch population, which was considered a large number of patients [50]. In literature, no threshold was found for a population size to have a profound impact on society. Therefore, we decided to look for a suitable analogy. A low impact on society can be expected for a small number of patients, such as in rare diseases. Although no single cut-off number has been agreed upon for rare diseases, they were defined by the European Commission on Public Health as affecting fewer than 1 in 2000 people, or 0.05% of the population [51]. We estimated that a 10-fold higher number of users, 1 in 200 people or 0.5% of the population, would be considered a large population which may have a profound impact on society.

### Impact

Certainly, the availability and characteristics of the alternative product are likely to influence the impact, but other elements can also contribute considerably to the impact of a specific shortage. In the levothyroxine case, for example, an alternative product with the same active substance was available. This shortage became unforgiven due to the combination of the lack of trust among patients toward the alternative product, the number of patients affected and the extra control of efficacy. In the epinephrine case, an alternative product with the same active substance as another brand was also available. This shortage became unforgiving due to the combination of the disease’s likelihood of fatality and the different usage instructions, which are of paramount importance for adequate treatment. The direct and indirect patient impact of shortages is determined by all five elements. The visualisation with radar charts clearly shows the impact of the shortages on the different elements and their different patterns. These different patterns underline that shortages cannot be regarded as a uniform phenomenon, even when they are all considered unforgiving.

### ECHO model

After identifying the elements, we observed a significant resemblance to the economic, clinical and humanistic outcomes (ECHO) model [52]. Besides the clinical outcomes, this model takes economic and humanistic outcomes into consideration. It is used to balance outcomes to ensure that a single outcome type is not being maximised at the expense of another type of outcome. Each ECHO outcome is represented by one or two of our elements. Economic outcomes are represented by the following elements: costs and number of patients affected. Clinical outcomes are represented by the following elements: disease, alternative product (primary aspects) and susceptibility (vulnerability). Humanistic outcomes are represented by product (secondary aspects) and by susceptibility. For shortages, the same three types of outcomes apply, and if one of them is not sufficiently met, the impact of a shortage is high. A recent scoping review on medicine shortages also categorised outcomes according to the ECHO model [53]. The ECHO model is used to balance outcomes. Therefore, we did not weigh the elements of impact.

### Limitations and strengths of the framework

Our framework faces three limitations. First, we rated the impact for the Dutch situation, which might differ from other countries for the same shortage. Differences in national systems, such as a national insurance system or the (in) ability to organise an alternative product such as a compounded pharmacy preparation, may cause a different impact of shortages on patients. We expect that at an international level, the same five elements would be identified, but perhaps impact rates would be scored differently. Second, we rated the impact at the start of the shortage. However, during a shortage, the elements might change mainly due to the extent to which an alternative product is available, resulting in a dynamic impact over time. In the penfluridol case, after 4 months, a compounded pharmacy preparation became available as an unlicenced product for on-label use. Patients and healthcare professionals considered this shortage to be resolved, whereas authorities and MAHs perceived this differently. This pharmacy preparation also increased the medicine costs by 540%. Because we rated the impact at the start of the shortage, we restrained ourselves to extra medicine and personnel costs. In addition, we have not taken the total costs of care into consideration, which can only be calculated retrospectively. In the situation of the BCG shortage the total costs of care of medium-risk and high-risk non-muscle-invasive bladder tumours doubled [54]. These aspects can be easily incorporated into the five elements we have identified. Third, we have rated our five learning cases on the population-wide level, and this may differ from an individual level. For instance in the doxycycline case, for most indications, another antibiotic was a suitable solution. For Q fever, however, doxycycline is the medicine of choice. Also, impact of costs may depend on the socioeconomic position of a patient, e.g. when a medicine is only partially reimbursed. Our framework can be easily adapted to be used for separate indications or subpopulations.

This framework is, as far as we know, the first systematic framework to take the elements of patient impact of a shortage from different stakeholders’ perspectives into account. These elements can have a direct impact on patients (such as Alternative product and Costs to patients) or an indirect impact (Costs to society) and were used as proxies for the patient perspective. This framework gives insight into the influence of these different elements. The insight may help stakeholders take quick, informed action on unforgiving shortages and thus mitigate the impact on patients. This framework also provides an opportunity for stakeholders to create a complete overview, to speak the same language and, ultimately, mitigate the impact of shortages for patients. We presented practical and recognisable examples to give direction to the application of this framework and to enhance its use. What this paper adds is a thinking framework for assessing the impact of medicine shortages in a systematic and patient-centred way. This framework also enables policymakers, regulators and health care providers to prioritise and allocate resources to support the needs of the clinic swiftly and efficiently. Medicine shortages are more than crude administrative or logistic numbers; they are about qualifying and estimating patient impact.

The COVID-19 pandemic causes medicine shortages. Medicines authorities across the world have developed and implemented an array of preparedness strategies [55, 56]. We see frameworks to mitigate medicine shortages that were rather unthinkable before the pandemic; for example, the approval of the veterinary drug propofol for use in intensive care units [57] or for lengthening expiration dates of certain critical products [58].

We focussed our framework on five learning cases and assessed data saturation with five more test cases. More research is needed to validate this framework. First, we focussed for this framework on direct and indirect impact. With the broader definition of impact [7], (un) intended and primary or secondary impact can be studied. Second, this framework was not reflected upon by key stakeholders to collect patient and general public perspectives. Methods on other health care related topics have been published [59, 60]. By applying our framework more broadly, aspects can be further refined, and triggers for the degree of forgiveness can be quantified.

## Conclusion

We created a framework for assessing the impact of a medicine shortage. We identified five key elements as core drivers behind the impact of a medicine shortage on patients, namely, alternative product, disease, susceptibility, costs, and number of patients affected. By rating these elements, an indication of the degree of forgiveness of a shortage can be obtained. More research is needed to validate our framework, and more insight into the impact which different elements have may help stakeholders quickly react to unforgiving shortages and thus mitigate their impact on patients.

## Supplementary Information

**Additional file 1. **Supplement – Impact of medicine shortages on patients: application on learning cases. This supplement provides details on the learning cases and the application of the framework.

## Data Availability

The datasets for this manuscript are not publicly available, since data is related to specific products and manufacturers, which is confidential information. Data on costs for medicines as well as age and number of users are third-party data. These data are not owned by the authors and belong to the Dutch Foundation for Pharmaceutical Statistics (SFK). The SFK welcomes applications from researchers for access to data. Applications are considered by the SFK Supervisory Board. Application forms for data access and further information are available at https://www.sfk.nl/english/foundation-for-pharmaceutical-statistics.
