# Supplementary material for: Impact of medicine shortages on patients - a framework and application in the Netherlands
Source: BMC Health Serv Res. 2022 Nov 17;22:1366. doi: 10.1186/s12913-022-08765-x (PMC9670055; doi:10.1186/s12913-022-08765-x)
Supplement: Supplementary file 1 — Additional file 1. Supplement – Impact of medicine shortages on patients: application on learning cases. This supplement provides details on the learning cases and the application of the framework. [file 12913_2022_8765_MOESM1_ESM.docx]

# Impact of medicine shortages on patients: application on learning cases

Doerine J. Postma^1,2^, Peter A. G. M. De Smet ^3^, Kim Notenboom^4^, Hubert G. M. Leufkens^1^, Aukje K. Mantel-Teeuwisse^1^

1. Division Pharmacoepidemiology & Clinical Pharmacology, Utrecht Institute for Pharmaceutical Sciences (UIPS), Utrecht University, the Netherlands
2. Royal Dutch Pharmacists Association, The Hague, the Netherlands
3. Radboud University Medical Centre, Radboud Institute for Health Sciences, Departments of IQ healthcare and of clinical pharmacy, Nijmegen, the Netherlands
4. Dutch Medicines Evaluation Board, Utrecht, the Netherlands

# Supplementary information

We applied our approach on the learning cases at the beginning of the shortages. We used information from KNMP Farmanco on possible solutions for patients for the alternative product, the patient information leaflet from the authorities for the disease as well as the use in vulnerable populations and the paper by Salomon[1] for the disability weight. The Dutch Foundation for Pharmaceutical Statistics (SFK) supplied data on the age of users, the costs for medicines and the number of users. The personnel costs were qualitatively determined from the case description. The determined rates for each element for the five learning cases are presented in Table 2.

## BCG instillation

An intravesical instillation with medicine from BCG is the most effective treatment for preventing the recurrence of non-muscle-invasive bladder tumours[2]. BCG instillations are produced worldwide by three manufacturers. Due to a production stop of one of the manufacturers, demand for the BCG instillations for the two remaining manufacturers increased and resulted in a shortage. The two remaining marketing authorisation holders (MAHs) tried to increase production to meet the increased demand, but one MAH experienced technical problems. It had to halt production due to potential contamination with other microorganisms[3]. The other MAH thus received an even higher increased demand and tried to keep up with this demand. They failed in meeting the demand quickly enough; it takes at least three months to boost production since this is the duration needed to grow a ‘batch’ of the microorganism BCG[4]. Despite all efforts, there was not enough supply available.

During this period, urologists inevitably had to stop BCG therapy or reduce the regular BCG dose to one-third and supplement it with alternative treatments (e.g., gemcitabine or mitomycin). The choice for the alternative treatment was based on the stage of the tumour, risk factors and anamneses[5]. This shortage was considered unforgiving because patients received a second-best treatment for a life-threatening indication such as cancer. This shortage led to agitation among patients and healthcare professionals. Based on information in the media and several fora at that time, patients feared the adjustment in their treatment might lead to the recurrence of cancer. Healthcare professionals were frustrated because of the lack of an equivalent alternative and the uncertainty about the arrival of new supplies.

### Scoring

No adequate alternative product was present for Bacillus Calmette-Guérin (BCG) instillation. Patients were inevitably treated with a different substance (licensed off-label gemcitabin or licensed on-label mitomycin), a third of the dose (off-label since this dose is not in accordance with the label) or no treatment at all. Since patients were mainly between 60 and 80 years, we consider them in general younger than 75 years[6]. These patients had no trust in this alternative treatment as for example expressed on internet fora. BCG is the primary treatment of bladder cancer, with a disability weight of 0.288. In the Netherlands 0.03% (4,600 patients) of the population have non-muscle-invasive bladder tumours[6] and since BCG is the preferred treatment we consider this the number of affected patients. The medicines costs were fully reimbursed and were lower for the alternative therapy than for the original treatment: from no treatment (0% of the costs of the BCG), gemcitabin (8% of the costs of the BCG) to mitomycin (59% of the costs of BCG). No signals were received on increased personnel costs needed to enable treatment switch.

## Doxycycline tablet

Doxycycline is a commonly prescribed antibiotic used to treat many different types of infection, such as bacterial pneumonia, acne, gonorrhoea, early Lyme disease and Q fever. During a shortage of doxycycline in 2013 caused by one MAH experiencing production problems[7], patients were treated with other antibiotics such as amoxicillin, azithromycin or erythromycin, depending on the indication.

This shortage had a significant impact on (a small number of) patients with Q fever and healthcare professionals. Physicians were unable to prescribe their first-choice treatment, doxycycline, to patients with Q fever. This caused commotion because of the uncertainty of the effectiveness of alternative treatments. Healthcare professionals had to organise an alternative treatment for this commonly prescribed antibiotic. Since most prescribers were not aware of the shortage, pharmacies had to contact each prescriber to discuss the alternative therapy, which took up a significant amount of healthcare professionals’ time. Both the inaccessibility of the preferred treatment and the time investment by healthcare professionals made this shortage unforgiving.

Scoring

The alternative for doxycycline was another licensed on-label antibiotic, such as amoxicillin, azithromycin or erythromycin with a different dose regimen. These alternatives caused extra medicines costs for amoxicillin (about 200% of the price of doxycycline), azithromycin (about 350% of the price of doxycycline) and erythromycin (about 1.300% of the price of doxycycline). Doxycycline is prescribed for moderate infectious diseases which have a disability weight of 0.051. These patients have in general no characteristic of vulnerability. Doxycycline would have been prescribed about 120,000 times during the shortage, affecting 0.71% of the Dutch population. Organising an alternative therapy for this large number of patients affected was time-consuming and caused increased personnel costs to society.

## Epinephrine auto-injector

Intramuscular injection of epinephrine by an auto-injector is used as a first-aid measure while waiting for medical assistance in case of anaphylaxis. Different brands of epinephrine auto-injectors have their own unique instructions for administration. A recall of batches of inoperable auto-injectors led to shortages and device switching several times over the last few years[8]. On each occasion, the alternative product was a different brand of licenced epinephrine auto-injector which was directly available. The availability of the different brands was closely monitored to ensure patients had access to an epinephrine auto-injector.

For proper use of the auto-injector, training the user or carer (e.g., family members or teachers) is crucial. Because of the uncertainty as to whether the injector delivers the medicine into the muscle layer, the European Medicines Agency (EMA) recommends patients carry two auto-injectors at all times[9].

During these shortages, three concerns came into view, resulting in an unforgiving shortage. First, the availability of the auto-injectors had to be guaranteed because of the life-threatening indication. Second, patients and carers had to be trained to use a different auto-injector brand since administration into the muscle layer is of paramount importance for the patient to respond to the treatment. Third, since 2015, patients have had to carry two epinephrine auto-injectors. In case of a shortage, patients may potentially be carrying two different brands with two different sets of instructions for administration, which possibly confuses the (stressed) user or carer who must concentrate on using the auto-injectors.

Scoring

The alternative product for the epinephrine auto-injector was another licensed epinephrine auto-injector. This alternative had a different instruction for administration resulting in moderate trust in the alternative and extra personnel costs to train users and carers. The alternative auto-injector was equally priced to the original, so medicines costs were equal. The auto-injector is used for anaphylaxis, which is life-threatening, resulting in a disability weight of 1. In the Netherlands 60,000 patients have an epinephrine auto-injector, which is 0.35% of the Dutch population. The patients using an auto-injector are adults and children (lower strength) between the age of approximately 3 and 9 years.

## Levothyroxine tablet

Levothyroxine tablets are used for hypothyroidism when the thyroid gland does not produce enough of this hormone to regulate the body’s energy and metabolism. Hypothyroidism is a disorder which affects between 0-3% and 3-7% of the population in the US and between 0-2% and 5-3% in Europe, depending on the definition used[10].

The MAH with the largest market share in the Netherlands had production problems, which created a shortage at the beginning of 2016. During the shortage, patients had to switch to another brand of levothyroxine tablets. Despite the fear that patients would not have access to levothyroxine, this did not happen because of the importation of extra products from other countries. Patients were unwilling to switch since they feared a severe change in their physical condition. They were also unwilling because of the extra costs due to an extra control of their blood levels after 6 weeks[11].

This shortage created a lot of commotion due to the large patient population, the consequences for patients when blood levels were suboptimal, the large market share of the product by one MAH and the late notification by that MAH. These aspects made this shortage unforgiving.

Scoring

The alternative product for levothyroxine tablet was another brand of authorized levothyroxine. For this alternative extra monitoring was necessary which created extra costs to patients. The price of another brand was equal or lower compared to the product in shortage. Patients had no specific characteristic of vulnerability but were very skeptical about another brand because of the fear of change in their condition. The disability weight for hypothyroidism is 0.019. This shortage affected 350,000 patients thus 2.06% of the Dutch population.

## Penfluridol tablet

Penfluridol tablets are used to treat patients with acute and chronic psychoses[12]. The tablets are administered once a week.

During the shortage in 2012, again due to production problems, a parenteral administration of long-acting antipsychotic medication was not considered an equivalent alternative since most patients reject injections. Also, a direct switch to other parenterally administered long-acting medication increases the risk of problems regarding efficacy and side effects. Inevitably, the pragmatic advice from experts was to administer oral pimozide, for which no evidence of effectiveness was available in the literature. Oral pimozide was also not regarded as an equivalent treatment since the intake frequency, every second day, was considered problematic for these vulnerable and often nonadherent patients. Eventually, an oral once-weekly administered pharmacy preparation containing penfluridol became available and was reimbursed.

This shortage occurred for medicine which had no equivalent alternative treatment available. This became very clear in 2009; penfluridol was deemed to be withdrawn from the market when the MAH announced they would stop the international production and marketing of penfluridol. Various stakeholders, including healthcare professionals and authorities, tried to keep penfluridol available, and as a result, another MAH took over the marketing authorisation.

It is well-known that a switch in therapy is especially difficult for medicines acting on the central nervous system since individual patients respond to specific medicines differently. Additionally, psychiatric patients are known for their reluctance to change. Patients treated with penfluridol often rely on community care services since they have trouble taking care of themselves. The vulnerability of the patients in combination with the absence of an adequate alternative made this shortage unforgiving.

Scoring

For penfluridol tablets no equivalent alternative was present. Inevitably, patients were treated with oral pimozide with a licensed product but off-label since this dose regimen is not in accordance with the label. The costs for the treatment increased around 250% with pimozide and were fully reimbursed. The indication residual schizophrenia has a disability weight of 0.558. Patients with schizophrenia usually depend on social care. They are in general very sceptical towards their therapy and have no trust in changes in their therapy. Penfluridol was used by about 13,000 patients, 0.08% of the Dutch population.

# References

1. Salomon JA, Vos T, Hogan DR, Gagnon M, Naghavi M, Mokdad A, Begum N, Shah R, Karyana M, Kosen S *et al*. Common values in assessing health outcomes from disease and injury: disability weights measurement study for the Global Burden of Disease Study 2010. Lancet (London, England) 2012; 380(9859):2129-2143.

2. Dal Moro F. BCG shortage in Europe. Preventive Medicine 2013; 57(2):146.

3. Messing EM. The BCG shortage. Bladder Cancer 2017; 3(3):227-228.

4. Colombel M. Socio-economic impact of BCG shortage. European Urology Supplements 2018; 17(1):60-62.

5. Veeratterapillay R, Heer R, Johnson MI, Persad R, Bach C. High-Risk Non-Muscle-Invasive Bladder Cancer—Therapy Options During Intravesical BCG Shortage. Current Urology Reports 2016; 17(9):68.

6. Dutch Cancer Society (KWF Kankerbestrijding), Netherlands Comprehensive Cancer Organisation (IKNL) D, Dutch Federation of Cancer Patientorganisations (NFK). Overige cijfers niet-spierinvasieve blaaskanker [Other facts on non-muscle-invasive bladder cancer]. <https://www.kanker.nl/bibliotheek/niet-spierinvasieve-blaaskanker/wat-is/11754-overige-cijfers-niet-spierinvasieve-blaaskanker>. Accessed:

7. Carris NW, Pardo J, Montero J, Shaeer KM. Minocycline as A Substitute for Doxycycline in Targeted Scenarios: A Systematic Review. Open forum infectious diseases 2015; 2(4):ofv178-ofv178.

8. Oxtoby K. All unexpired Emerade autoinjectors recalled after pens fail to activate.

Pharmaceutical Journal 2019.

9. European Medicines Agency. Better training tools to support patients using adrenaline auto-injectors. 2015. <https://www.ema.europa.eu/documents/referral/adrenaline-auto-injectors-article-31-referral-better-training-tools-recommended-support-patients_en.pdf>. Accessed: 15 September 2022

10. Chaker L, Bianco AC, Jonklaas J, Peeters RP. Hypothyroidism. Lancet

2017; 390(10101):1550-1562.

11. Fliers E, Demeneix B, Bhaseen A, Brix TH. European Thyroid Association (ETA) and Thyroid Federation International (TFI) Joint Position Statement on the Interchangeability of Levothyroxine Products in EU Countries. Eur Thyroid J 2018; 7(5):238-242.

12. Soares BGO, Silva de Lima M. Penfluridol for schizophrenia. Cochrane Database of Systematic Reviews 2006; (2).
